# A Rapid and Adaptive Alignment under Mooring Condition Using Adaptive EKF and CNN-Based Learning

**DOI:** 10.3390/s20154069

**Published:** 2020-07-22

**Authors:** Jong Nam Lim, Chan Gook Park

**Affiliations:** Department of Aerospace Engineering, Seoul National University, Seoul 08826, Korea; ljn78@snu.ac.kr

**Keywords:** convolution neural network (CNN), adaptive extended Kalman filter (EKF), alignment, mooring environment, inertial navigation system (INS)

## Abstract

Alignment of the inertial navigation system (INS) in the mooring environment should take into account the movements of the waves or wind. The alignment of the INS is performed through an extended Kalman filter (EKF) using zero velocity as a measurement. However, in the mooring condition, this is not perfect stationary, thus the measurement error covariance matrix should be adjusted. In addition, if the measurement error covariance matrix is fixed to one value, the alignment time may take longer or the performance may be reduced depending on the change in mooring conditions. To solve this problem, we propose an alignment method using adaptive Kalman filter and convolution neural network (CNN)-based learning. The proposed method was verified for the superiority of alignment time and accuracy through Monte Carlo simulation in a mooring environment.

## 1. Introduction

The inertial navigation system (INS) is the most fundamental navigation system that provides position, velocity, and attitude information using the gyro to measure angular velocity and the accelerometer to measure the linear acceleration of the vehicle. The strapdown-based INS calculates navigation solution in the navigation frame from the output of the inertial sensors attached to the body frame through recursive integration and coordinate transformation based on initial information. Therefore, it is necessary to find the initial attitude precisely, and this process is called alignment [1,2].

Alignment in the stationary state is performed using the Earth’s rate and gravitational acceleration. To perform the alignment, a gyrocompassing method using a control law or an estimator based on a Kalman filter technique capable of estimating errors of sensors as well as an attitude during alignment has been applied.

The stationary state has a complete zero velocity, thus the alignment process is performed using the zero-velocity information as a measurement in Kalman filter. However, if the alignment is performed in a sea environment, the velocity and attitude are continuously changed by the wind and wave. Therefore, it is essential for navigation systems operated on marine platforms, such as a ship and a submarine, to apply the technique of performing alignment in sea environment [1,2,3].

We conducted a study on Extended Kalman Filter (EKF)-based alignment using INS in a mooring environment. Here, EKF, which is a linearized Kalman filter, is applied with consideration to the integration with the nonlinear system INS. The most crucial point is how to handle this linear velocity and rotation that repeatedly occur in the mooring environment with wind or wave. These movements appear as errors or disturbances that interfere with a stationary condition, thus EKF-based alignment can be performed by setting the measurement error covariance large.

However, in the continuously changing mooring environment, this method may degrade performance depending on changing situations and takes a long time to finish the alignment. Therefore, we propose adaptive methods using adaptive EKF and CNN-based learning for alignment in the mooring environment. The adaptive EKF uses innovation of the measurement to estimate the measurement error covariance matrix continuously, and outputs an adaptive result according to the condition of the measurement. The CNN-based learning method learns the optimal measurement error covariance matrix to minimize the error in changing the mooring environment based on a large number of data.

## 2. Related Work

Various methods of using digital filters such as FIR or IIR have been developed to deal with this problem. Lian et al. proposed a method to reduce the effect of acceleration disturbance by using a finite impulse response (FIR) filter [4]. Feng et al. applied the infinite impulse response (IIR) filter and power spectrum analysis to compensate for the disadvantages of the FIR filter that must have a huge order [5]. However, this digital filter method has a delay problem in real-time applications and has limitations due to a fixed filter coefficient in a sea environment in which the magnitude or period of motion continuously changes.

Gaiffe et al. proposed an inertial frame-based alignment (IFBA) technique to project gravity on an inertial frame to estimate the coordinate transformation matrix between the inertial frame and the body frame [6]. Since then, this IFBA technique has been used by many researchers for alignment in mooring [5,7,8]. Gao et al supposed a fast alignment method using IFBA, and bidirectional Kalman filter [7], and Sun et al applied the IFBA and hidden Markov model to prevent filter delay for alignment in mooring [8].

If the conventional EKF is used for alignment in the mooring environment, the alignment is disturbed by rate and moving. Therefore, it is necessary to set an appropriate error covariance, and the time required for alignment is increased. The adaptive EKF has been applied to various cases in which the environment or condition changes. It is classified into a case where the measurement changes and a case where the system model changes [9,10]. The alignment in mooring uses zero velocity as a measurement, and since the disturbance caused by the waves or wind can be regarded as an error in the measurement, we use a measurement dependent adaptive EKF.

The deep learning technique has been widely used in fields that solve classification, recognition, and estimation problems in various nonlinear environments using many data. Among various deep learning techniques, CNN was first introduced by Lecun and applied to handwriting zip code recognition. Then, it was generalized and expanded in concept, making it widely used in image processing, recognition, and classification [11,12,13].

Recently, research to apply deep learning techniques to the Kalman filter has been conducted. In the process of applying the Kalman filter to the pose estimation problem, Coskun et al. proposed a long short-term memory–Kalman filter (LSTM-KF) that learn motion model, measurement model and noise covariance of Kalman filter through three independent LSTM modules [14]. Martin proposed a method to reduce navigation errors when driving a car by learning [15].

Lee and Bang calculated the filter coefficient of RBPF in terrain-aided navigation (TAN) through the LSTM-based learning [16]. By estimating the measurement error covariance, system error covariance, and measurement model using four LSTM modules, it showed high performance in the TAN environment with strong nonlinearity.

## 3. Conventional Extended Kalman Filter Based Alignment in Mooring Environment

Since alignment is performed based on INS, the model of INS is used as the system model, and the zero-velocity model is used as the measurement model for the Kalman filter. Furthermore, we apply an indirect method based Conventional Extended Kalman filter (CEKF) that linearizes the Kalman filter for the nonlinear INS model.

As mentioned above, the easiest Kalman filter-based method to perform alignment in the mooring environment is to set the filter coefficients with consideration of movement conservatively. When the zero velocity is used as a measurement in the mooring environment, repetitive changes in velocity or attitude due to wind or waves occur in the form of a sin wave. Since this can be regarded as the large error of the measurement, the Kalman filter should be performed by setting the measurement error covariance matrix R to be larger than the conventional case.

The conventional EKF recursively performs the measurement update and time propagation. The nonlinear system model and measurement model are as follows.

(1) $$\dot{x}(t)=f\left(x(t)\right)+w(t)$$

(2) y(t)=h(x(t))+v(t)

(3) w(t)~N(0,   Q),v~N(0,   R)

where f is a nonlinear function for the system equation; h is a nonlinear function for the measurement equation; x is a state variable; y is a measurement; w is a noise of the system model; v is noise of the measurement model, which follows a Gaussian Probability Density Function (PDF); Q is system error covariance matrix; and R is measurement error covariance matrix.

The results of linearizing and discretizing Equations (1) and (2) and expressing them in error state are as follows [1,3].

(4) $$\delta {\hat{x}}_{k+1}=(I+F_{k}\Delta t)\delta {\hat{x}}_{k}+w_{k}$$

(5) $$\delta y_{k}=H_{k}\delta {\hat{x}}_{k}+v_{k}$$

where

(6) $$F_{k}={\frac{\partial f_{k}}{\partial x_{k}}|}_{x_{k}={\hat{x}}_{k}},\;\;\;H_{k}={\frac{\partial h_{k}}{\partial x_{k}}|}_{x_{k}={\hat{x}}_{k}}$$

The time update equation of EKF is as follows.

(7) $$\delta {\hat{x}}_{k+1|k}=F_{k}\delta {\hat{x}}_{k}$$

(8) $$P_{k+1|k}=F_{k}P_{k}^{}F_{k}^{T}+Q_{k}$$

The measurement update equation of EKF is as follows.

(9) $$\delta {\hat{x}}_{k+1}=\delta {\hat{x}}_{k+1|k}+K_{k}(\delta z_{k}-H_{k}\delta {\hat{x}}_{k+1|k})$$

(10) $$S_{k+1}^{}=H_{k+1}P_{k+1|k}^{}H_{k+1}^{T}+R_{k+1}$$

(11) $$K_{k+1}=P_{k+1|k}^{}H_{k+1}^{T}S_{k+1}^{-1}$$

(12) $$P_{k+1|k+1}^{}=P_{k+1|k}^{}-K_{k+1}S_{k+1}^{}K_{k+1}^{T}$$

The nonlinear error model of the INS system is as follows [1,2], and the system matrix $F_{k}$ refers to Appendix A.

(13) $$\begin{array}{l} \delta {\dot{V}}^{n}=\left[C_{b}^{n}f^{b}\right]\times \delta \Phi^{n}-\left[2\omega_{ie}^{n}+\omega_{en}^{n}\right]\times \delta V^{n}+C_{b}^{n}\delta f^{b}+V^{n}\times \left[\delta \omega_{en}^{n}\right]\\ \delta {\dot{\Phi }}^{n}=\delta \omega_{in}^{n}-\omega_{in}^{n}\times \delta \Phi^{n}-C_{b}^{n}\delta \omega_{ib}^{b}\\ \delta \dot{\nabla }=0\\ \delta \dot{\varepsilon }=0 \end{array}$$

(14) $$\delta x={\left[\begin{array}{cccc} \delta V^{n} & \delta \Phi^{n} & \delta \nabla & \delta \varepsilon \end{array}\right]}^{T}$$

where $\delta V^{n}$ is a velocity error, $\delta \Phi^{n}$ is an attitude error, δ∇ is an accelerometer error, δε is a gyro error, $C_{b}^{n}$ is a coordinate transformation matrix that converts from body frame to navigation frame, $\omega_{ie}^{n}$ is an angular rate due to the Earth rate in navigation frame, $\omega_{en}^{n}$ is an angular rate due to the transpose rate in navigation frame, $g^{n}$ is a gravity acceleration in navigation frame, and $f^{b}$ is a specific force in body frame.

(15) $$\begin{array}{l} \delta y_{k}=\left[\begin{array}{ccc} \delta {\hat{V}}_{k}^{n} & \delta {\hat{V}}_{k}^{e} & \delta {\hat{V}}_{k}^{d} \end{array}\right]\\ \delta z_{k}=H_{k}\delta x_{k}+v_{k}={\left[\begin{array}{ccc} \delta {\bar{V}}_{k}^{n} & \delta {\bar{V}}_{k}^{e} & \delta {\bar{V}}_{k}^{d} \end{array}\right]}^{T}={\left[\begin{array}{ccc} {\bar{V}}_{k}^{n}-0 & {\bar{V}}_{k}^{e}-0 & {\bar{V}}_{k}^{d}-0 \end{array}\right]}^{T} \end{array}$$

where $\delta {\hat{V}}_{k}^{n}$ is an error of propagated north-direction velocity, $\delta {\hat{V}}_{k}^{e}$ is an error of propagated east-direction velocity, $\delta {\hat{V}}_{k}^{d}$ is an error of propagated down-direction velocity. ${\bar{V}}_{k}^{n}$ is a measured north-direction velocity, ${\bar{V}}_{k}^{e}$ is a measured east-direction velocity, ${\bar{V}}_{k}^{d}$ is a measured down-direction velocity, $\delta {\bar{V}}_{k}^{n}$ is an error of measured north-direction velocity, $\delta {\bar{V}}_{k}^{e}$ is an error of measured east-direction velocity, and $\delta {\bar{V}}_{k}^{d}$ is an error of measured down-direction velocity.

However, in the case of using the fixed measurement error covariance matrix, performance and convergence speed of the Kalman filter change according to sea conditions, which change with time or position. In general, assuming that system error covariance matrix Q is appropriately fixed, if the measurement error covariance matrix R becomes small, the filter believes the measurement more. Since it is judged that the measurement is accurate, the performance is improved, and convergence speed is fast. However, if the error of the actual measurement is larger than the expected measurement error, performance of the filter degrades, and even the filter may diverge.

Conversely, if the measurement error covariance matrix R is large, the filter judges that the error of the measurement is significant. Thus, the convergence speed becomes slow, but the divergence of the filter can be avoided. In other words, if the error of the actual measurement in the mooring environment is similar to the error of the expected measurement, the best result is estimated. Since the error of actual measurement in the mooring environment is changing, conventional EKF using a fixed measurement error covariance matrix may degrade performance.

Therefore, we propose two methods for alignment under the mooring condition, as described in the next sections.

## 4. Adaptive EKF Based Alignment in Mooring Environment

The adaptive EKF is to adaptively change measurement error covariance matrix R using an innovation sequence, which is defined as the difference between the measured value and the estimated value. The innovation sequence is as follows [9].

(16) $$i_{k}=\delta z_{k}-H\delta {\hat{x}}_{k}=\delta z_{k}-HF\delta {\hat{x}}_{k-1}$$

where $i_{k}$ is an innovation sequence, $z_{k}$ is a measurement, H is a measurement model matrix, and F is a system model matrix. 

Substituting the measurement in Equation (15) into Equation (16),

(17) $$\begin{array}{l} i_{k}=\delta z_{k}-HF\delta {\hat{x}}_{k-1}=H\delta x_{k}+v_{k}-HF\delta {\hat{x}}_{k-1}\\ \;\;\;\;\;=H(\delta x_{k}-F\delta {\hat{x}}_{k-1})+v_{k}=H{\tilde{x}}_{k|k-1}+v_{k} \end{array}$$

Innovation sequence is a kind of indicator of real estimation errors that can be used for the adaptive algorithm. The covariance of innovation is calculated using the above equation and is as follows [9,10].

(18) $$I_{k}=E\left[i_{k}i_{k}^{T}\right]=HP_{k|k-1}H^{T}+R_{k}$$

Therefore, an adaptive measurement error covariance matrix is as follows.

(19) $$R_{k}=I_{k}-HP_{k|k-1}H^{T}$$

where $I_{k}$ is a covariance of innovation, $i_{k}$ is an innovation, H is an observation matrix, $P_{k|k-1}$ is a state error covariance, and $R_{k}$ is a measurement error covariance matrix.

In the stationary system and noise environment, $I_{k}$ can be calculated by using the average as following equation [17].

(20) $${\hat{I}}_{k}=\frac{1}{k}\sum_{m=1}^{k}i_{m}i_{m}^{T}$$

Moreover, converting the above equation to recurrent form is as follows.

(21) $${\hat{I}}_{k}=\frac{k-1}{k}{\hat{I}}_{k-1}+\frac{1}{k}i_{k}i_{k}^{T}$$

Substituting Equation (21) into Equation (19), we can obtain the estimated adaptive measurement error covariance matrix as follows.

(22) $${\hat{R}}_{k}={\hat{I}}_{k}-HP_{k|k-1}H^{T}$$

At this time, to prevent R from becoming negative in the case ${\hat{I}}_{k}$ is too small, the following equation is added.

(23) $$if\;diag\left({\hat{R}}_{k}\right)<0,\;\;then\;\;diag\left({\hat{R}}_{k}\right)=0$$

An innovation is obtained from the measurement, and the covariance of innovation is calculated through data. Since the calculated covariance of innovation represents the condition of the measurement, the measurement error covariance matrix is automatically adjusted according to the current measurement. In other words, if the wave is large, the measurement error is large, so the measurement error covariance matrix R is increased. Conversely, if the wave is small, the measurement error is small, and then the measurement error covariance matrix R is decreased.

## 5. Learning-Based Alignment in Mooring Environment

### 5.1. Introduction of CNN

In this section, the measurement error covariance matrix R is adjusted according to the wave condition using the trained network. In this paper, convolutional neural network (CNN) is applied to learn time-series sensor data. Recursive neural network (RNN) is generally used to process this kind of time series data. However, this time series data can also be processed as a convolution feature, thus this study uses a one-dimensional convolution network. Compared to RNN, CNN has the advantage of less complexity and reduced computation, and better extraction of data characteristics according to conditions. In fact, in the field of audio generation and machine translation, CNN has shown good results in the past few years. 

CNN extracts important characteristic among related data. In other words, CNN is not just learning a list of data as in the multi-layer perceptron (MLP) but learning spatial correlation by assigning local connections between neurons in the adjacent layer. CNN compresses the characteristics of the original signal through convolution. At this time, according to the filter used, a feature map is constructed to output only the characteristics that can best represent the data. The feature map starts from low-level feature information and synthesizes low-level features as the layer deepens to generate high-level features.

Learning using this CNN has the advantage of being able to process many data at a time through a convolution operation, and significantly reducing the number of parameters to be learned through the sharing of weights and the receptive field. In addition, CNN can extract and learn spatial and temporal characteristics of data through spatial/temporal correlation, thus it has a robustness against noise or external disturbance [18].

The input through sensors is continuous time-series data, and data of the previous time and current time are dependent and have a relationship by system model. 

### 5.2. Architecture of CNN

#### 5.2.1. Architecture 

**Figure 1:**
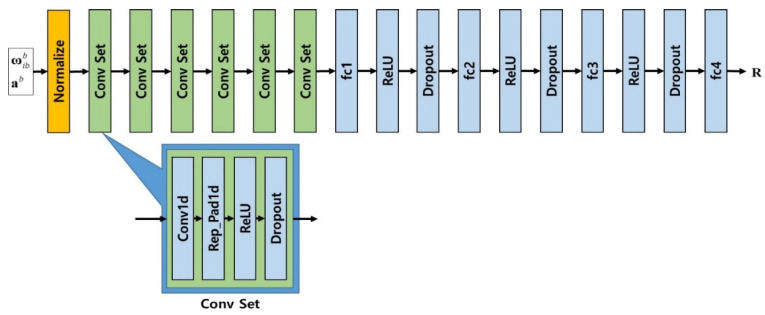
Architecture of CNN layers.

In this paper, CNN is composed of an input layer that receives sensor input, a convolution layer that performs synthesis, a ReLU activation function that adds nonlinearity, a dropout layer, and an output layer. The output layer consists of a fully connected layer and outputs the measurement error covariance matrix R, as shown in Figure 1.

$\omega_{ib}^{b}$ is an angular rate that is the output of gyro, $a_{}^{b}$ is a linear acceleration that is an output of accelerometer, and Conv Set is composed of Conv1d representing one-dimensional convolution layer, Rep_Pad1d, which creates one-dimensional padding by copying the boundary values, ReLU activation function, and Dropout layer. 

The input of learning is the output of the inertial sensors, which is linear acceleration and angular rate. Since the values have different characteristics, normalization preprocessing is performed using the average and variance of the sensor output. The output is measurement error covariance matrix R, which is the parameter that controls the EKF. 

#### 5.2.2. Input Data and Normalization

The network inputs consist of three-axis gyros and accelerometers. When performing alignment during mooring, the ship moves repeatedly depending on the size of the sea waves, so the parameter of the filter must be adjusted according to this movement. Gyros measure rotation and accelerometers measure linear acceleration, thus the networks input is the physical quantity measured by the gyros and accelerometers. 

The scale of the physical quantity measured by the gyros and accelerometers is not the same. In the static condition, the acceleration on the horizontal x and y axes is not large. However, the z axis accelerometer measures the Earth’s gravitational acceleration, thus it outputs a relatively large value. In addition, the gyros measure the Earth rate, but its size is very small. 

Therefore, normalization is performed to make the range of all physical quantities equal. Normalization is performed using the mean and standard deviation as shown in Equations (24)–(27).

(24) $$u_{n}=(u-u_{mean})/u_{std}$$

where

(25) $$u={\left[\begin{array}{ccccccc} \omega_{ib}^{bx} & \omega_{ib}^{by} & \omega_{ib}^{bz} & | & a_{}^{bx} & a_{}^{by} & a_{}^{bz} \end{array}\right]}^{T}$$

(26) $$u_{mean}=\frac{1}{N}\sum_{i=1}^{N}u^{\left(i\right)}$$

(27) $$u_{std}=\sqrt{\frac{1}{N}\left[\sum_{i=1}^{N}{\left(u^{\left(i\right)}-u_{mean}\right)}^{2}\right]}$$

Since the calculation frequency of the inertial navigation and EKF filter is 10 Hz, the sampling time of the input data for training is 0.1 s. For continuous time series data to output the measurement error covariance matrix R, continuous sensor data of 20 s were used.

#### 5.2.3. Convolution Layer

One-dimensional convolution layer is applied to perform the convolution operation of time series sensor data. The output of the convolution layer is input to the ReLU activation function to add nonlinearity, and the dropout layer is connected to the activation function to reduce the size and give generality. Total convolution layers are composed of six consecutive Conv Sets.

**Figure 2:**
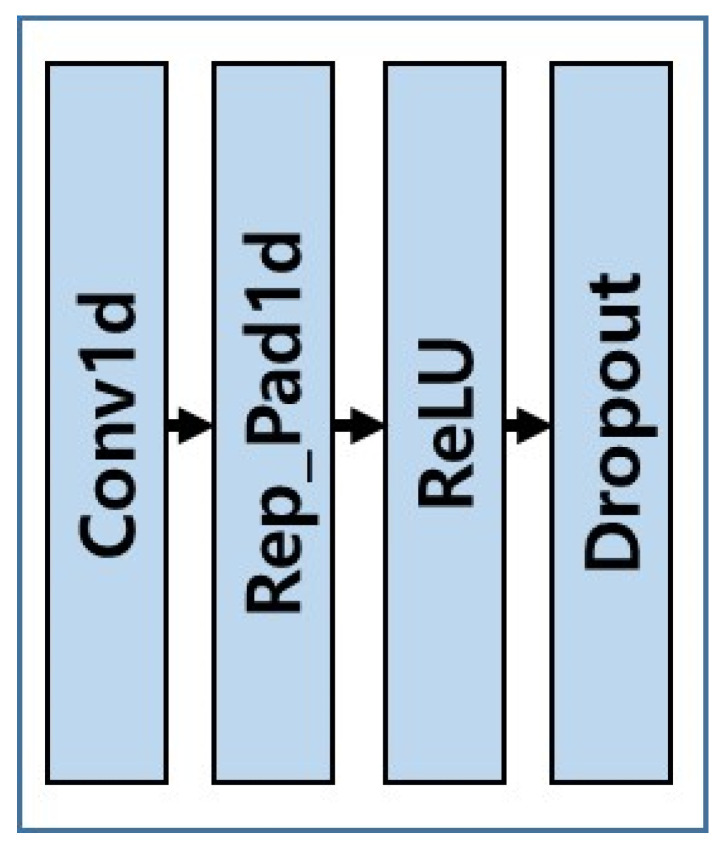
Block diagram of Conv Set.

One Conv Set is composed of one-dimensional convolution layer, one-dimensional padding layer, one ReLU, and one dropout layer, as shown in Figure 2.

In this paper, the conv1d function provided by PyTorch is applied to construct one-dimensional convolution layer; the length of output sequence is determined by Equation (28).

(28) $$L_{out}=\left[\frac{L_{in}+2\times P-D\times (K-1)-1}{S}+1\right]$$

where $L_{out}$ is the length of output sequence, $L_{in}$ is the length of input sequence, P is the length of padding, D is the length of dilation, K is the length of kernel (filter), S: length of stride.

The values of each parameter for CNN layer used here are shown in Table 1 and the length of the output sequence was designed to remain the same as the length of the input sequence.

#### 5.2.4. Fully Connected Layer and Network Output

The output of the convolutional layer is connected to the fully connected layers. The final output, the measurement error covariance matrix R, is calculated through the output of a fully connected layer. All fully connected layers are composed of four consecutive layer sets.

The output of the fully connected layer is connected to the ReLU activation function and goes through the dropout layer to add generality. The last fully connected layer omits the ReLU and dropout layer to output the measurement error covariance R.

The size of each fully connected layer is [200 × 2000], and this size remains the same in each fully connected layer. The output size of the final fully connected layer is [200 × 6]. The output value of network is finally converted to the measurement error covariance R using Equation (29). Here, dimension of R is [200 × 3].

(29) $$R=R_{0}\times 10^{z}$$

where R is the measurement error covariance matrix, $R_{0}$ is the initial measurement error covariance matrix, z is output of network.

#### 5.2.5. Loss Function

Mean squared error applies to the loss function. Since the measurement of the EKF becomes the zero velocity, the error is the difference between the velocity estimate and the true velocity, as shown in Equation (30).

(30) $$Loss\left(\gamma \right)=\frac{1}{n}\sum_{i=1}^{n}{\left\|{\hat{V}}_{i}^{n}\left(\gamma \right)-V_{i,True}^{n}\right\|}^{2}$$

where ${\hat{V}}_{i}^{n}\left(\gamma \right)$ is estimated velocity vector at time i, $V_{i,True}^{n}$ is true velocity vector at time i.

#### 5.2.6. Optimizer

The purpose of this learning is to optimize the measurement error covariance matrix R for EKF that minimizes the loss of Equation (30). This model updates the parameters using the gradient obtained through backpropagation. In this paper, PyTorch framework is applied as a tool for learning, and the optimizer used Adam optimizer that reflects the adaptive learning rate algorithm that combines the ideas of momentum optimization and RMSprop for fast and stable learning.

Adam optimizer use two parameters (learning rate and weight decay) for optimization. In this study, the learning rate of 0.00001 and the weight decay of 0.000001 were selected to satisfy the learning speed and accuracy.

#### 5.2.7. Parameter Tuning for CNN Architecture

If the length of this sequence is too long, the characteristics of the rapidly changing sea wave cannot be learned well. It is also known that the size of an excessively long input window can over-smooth the result [19]. Conversely, if the length of this sequence is too short, the coefficient of the covariance changes too quickly, which can degrade the stability of the filter. Therefore, considering the above characteristics, the input length of the sequence data for training was set to 20 s.

In this paper, the architecture of the overall filter is superposition on the Conv Set structure to which the one-dimensional filter of 5 × 1 size is applied. This repetitive placement of the Conv Set utilizes non-linearity thorough the ReLU block and Dropout block in the middle of the Conv Set, thus it can make the desired feature stand out more and is advantages in terms of computation amount.

Conv Set was designed by configuring filter block, padding block, and dilation block so that the length of the input sequence and the output sequence are the same to keep the computation constant and balance the system. That is, even after passing through one Conv Set, 200 (20 s × 10 Hz) sequence data maintain their length.

Learning parameters for CNN architecture are shown in Table 2.

## 6. Simulation 

### 6.1. Simulation Environment

**Figure 3:**
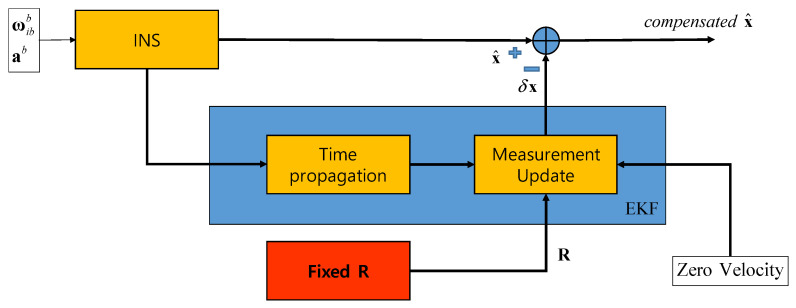
Test Case 1: CEKF with fixed R.

**Figure 4:**
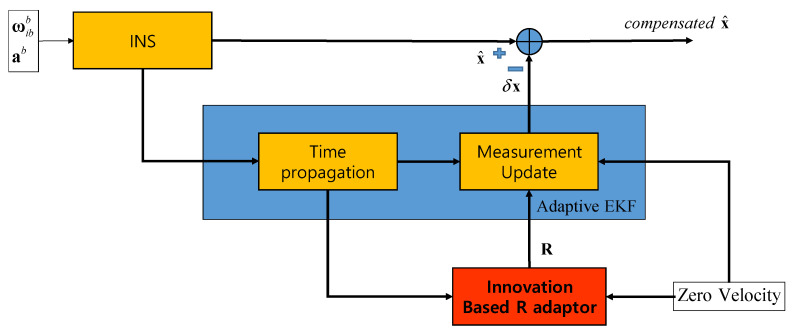
Test Case 2: Adaptive EKF.

**Figure 5:**
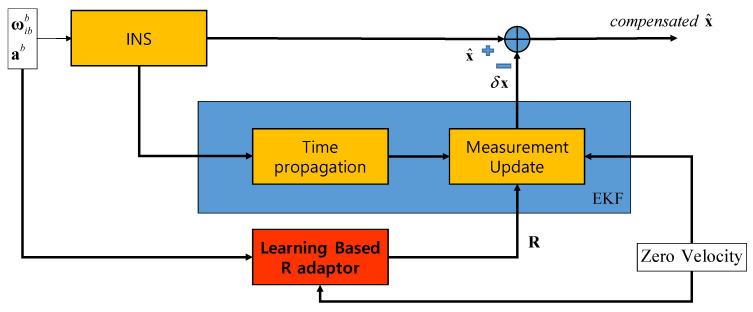
Test Case 3: Learning-based EKF.

To verify the performance of a proposed method, the mooring environment was simulated, and convergence speed and performance accuracy were compared by performing the alignment for the case of using a fixed measurement error covariance matrix, adaptive EKF, and learning as shown in Figure 3, Figure 4 and Figure 5.

Figure 3 shows the case of using a fixed error covariance R. INS uses the acceleration and angular velocity to calculate the navigation solutions. The EKF module located in the middle consists of the time propagation and measurement update process. The measurement update process is performed by applying the zero-velocity measurement and the fixed R value indicated in the red box. The compensated is obtained by subtracting the estimated error state from EKF to the navigation solution from INS.

Figure 4 shows the case where the adaptive EKF described in the Section 4 is used. The other parts are the same as in Figure 3. However, R is calculated adaptively in the red box labeled “Innovation Based R adaptor”. The calculated R is used in the EKF measurement update in EKF.

Figure 5 shows the case where R calculated by learning is used. The error covariance matrix R is calculated thorough the network updated by learning in the red box marked “Learning-based R adaptor”. The calculated R is used in the EKF measurement update in EKF.

To simulate the mooring, linear motion and angular rate are generated by the following equations and added to the gyro and accelerometer outputs.

(31) $$\phi =A_{\phi }\cos\left[\frac{2\pi }{T_{\phi }}t\right],\;\;\theta =A_{\theta }\cos\left[\frac{2\pi }{T_{\theta }}t\right],\;\;\psi =A_{\psi }\cos\left[\frac{2\pi }{T_{\psi }}t\right]$$

(32) $$P_{x}^{b}=A_{Pbx}\sin\left[\frac{2\pi }{T_{Pbx}}t\right],\;\;P_{y}^{b}=A_{Pby}\sin\left[\frac{2\pi }{T_{Pby}}t\right],\;\;P_{z}^{b}=A_{Pbz}\sin\left[\frac{2\pi }{T_{Pbz}}t\right]$$

Velocity outputs can be expressed as follows.

(33) $$V_{x}^{b}=A_{Pbx}\frac{2\pi }{T_{Pbx}}\cos\left[\frac{2\pi }{T_{Pbx}}t\right],\;\;V_{y}^{b}=A_{Pby}\frac{2\pi }{T_{Pby}}\cos\left[\frac{2\pi }{T_{Pby}}t\right],\;\;V_{z}^{b}=A_{Pbz}\frac{2\pi }{T_{Pbz}}\cos\left[\frac{2\pi }{T_{Pbz}}t\right]$$

where $A_{\phi },\;A_{\theta },A_{\psi }$ are the amplitude of attitude movement [roll, pitch, heading], $A_{Pbx},\;A_{Pby},\;A_{Pbz}$ are the amplitude of position movement [x, y, z], $T_{\phi },\;\;\;T_{\theta },\;\;\;\;T_{\psi }$ are the duration time of attitude movement [roll, pitch, heading], and $T_{Pbx},\;\;T_{Pby},\;\;T_{Pbz}$ are the duration time of position movement [x, y, z].

**Figure 6:**
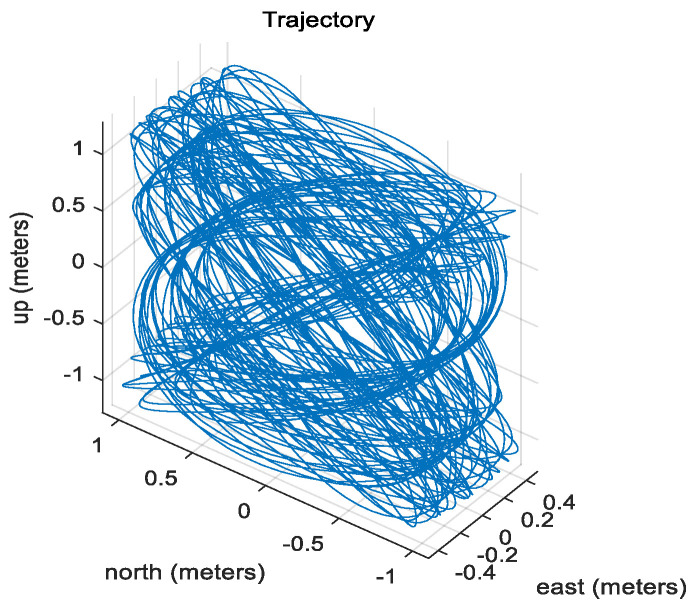
Trajectory in mooring environment.

**Figure 7:**
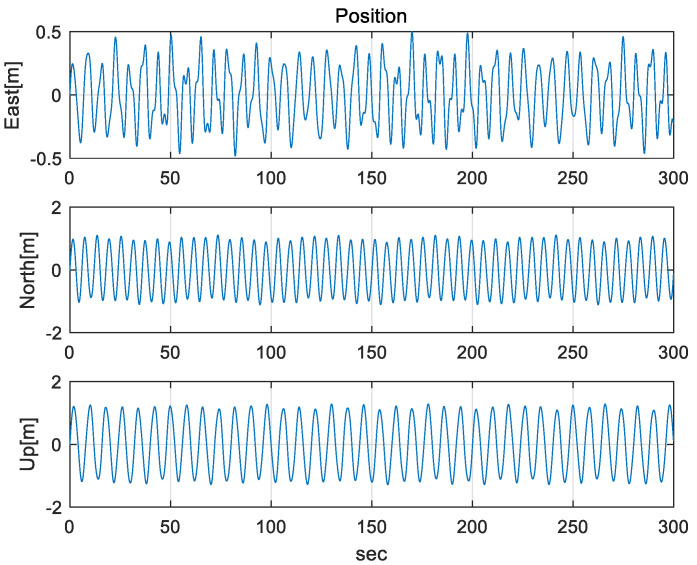
Reference Position in mooring environment. (Top: East-directional position, Middle: North-directional position, Bottom: Up-directional position).

**Figure 8:**
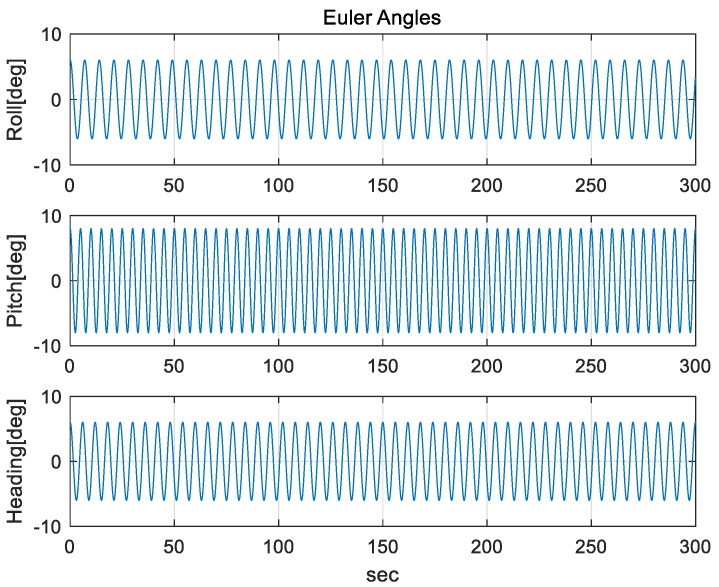
Reference Attitude in mooring environment. (Top: Roll angle, Middle: Pitch angle, Bottom: Heading angle).

**Figure 9:**
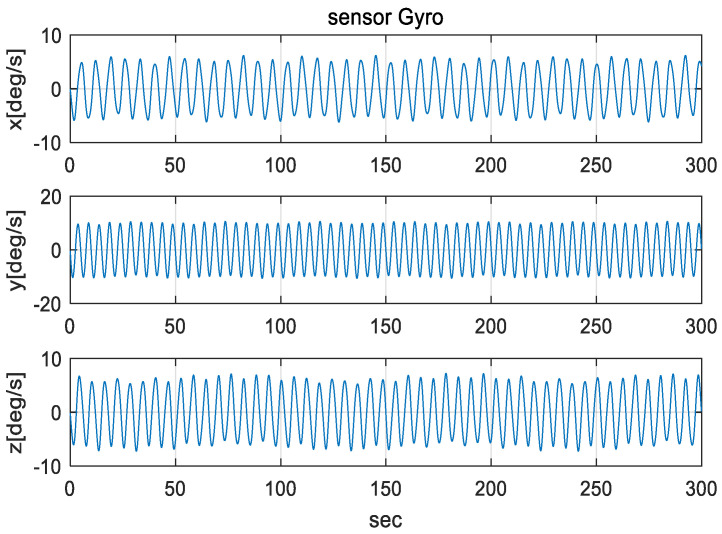
Output of Gyro Sensor in mooring environment. (Top: X-directional gyro output, Middle: Y-directional gyro output, Bottom: Z-directional gyro output).

**Figure 10:**
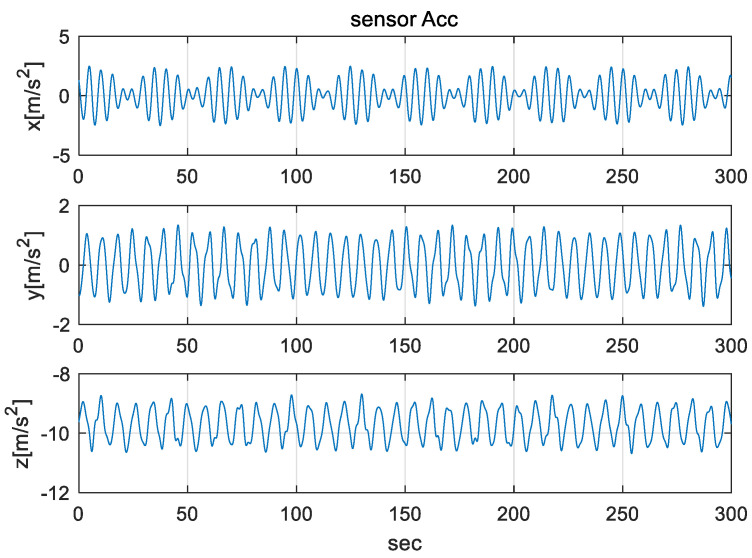
Output of Accelerometer in mooring environment. (Top: X-directional accelerometer output, Middle: Y-directional accelerometer output, Bottom: Z-directional accelerometer output).

The IMU sensors (gyros and accelerometers), initial navigation error, and parameters used in the simulation are shown in Table 3. To simulate the mooring environment, reference values for position, velocity, and attitude were generated using the parameter values defined in Table 3 and Equations (31)–(33). Figure 6 shows the position of a vehicle in the mooring condition. Figure 7 and Figure 8 show the reference position and attitude generated under mooring conditions. Figure 9 and Figure 10 show the true output of the gyro sensors and accelerometers sensors generated under mooring conditions.

Input data for learning were randomly generated using the values in Table 3. Based on the same reference trajectory, sensor errors, initial navigation errors, and mooring conditions were changed. The inertial sensor measurements were generated by randomly defining bias and noise with values that follow a normal distribution. In the same way, the initial navigation errors were generated by randomly defining the initial position errors, initial velocity errors, and initial attitude errors with values that follow a normal distribution. The mooring condition was generated by randomly defining the size and duration time of waves with values follow uniform distribution. The network was trained using the generated input data, and the training was repeatedly performed up to the maximum epoch, as defined in Table 2 for each data sequence.

The simulation was performed by dividing the mooring condition into three movements: (1) small size of movement; (2) medium size of movement; and (3) large size of movement. As shown in Table 3, the small size of the movement is defined as 10% of the amplitude of the large size of movement, and medium-size movement is defined as 50% of the amplitude of the large size of movement. Alignment is performed in each case to compare the alignment accuracy and speed according to the method of applying the measurement error covariance matrix. As described above, it is divided into a method using CEKF with a fixed measurement error covariance matrix R, a method using adaptive EKF, and a method using CNN-based learning.

For coarse alignment, a feedback-based alignment technique can generally be applied in a mooring environment [9,10]. However, this paper focuses on fine alignment using Kalman filter and zero-velocity measurement, assuming that coarse alignment has been performed.

In consideration of the mooring environment, the initial attitude error after coarse alignment was set to 0.1° (roll/pitch, 1 σ) and 5° (heading, 1 σ). To perform the Monte Carlo simulation, sensor errors and initial errors were randomly generated. Repeated simulation was performed 100 times in each case, and the results were analyzed for the RMS error, alignment speed, and final error values.

### 6.2. Simulation Results

**Figure 11:**
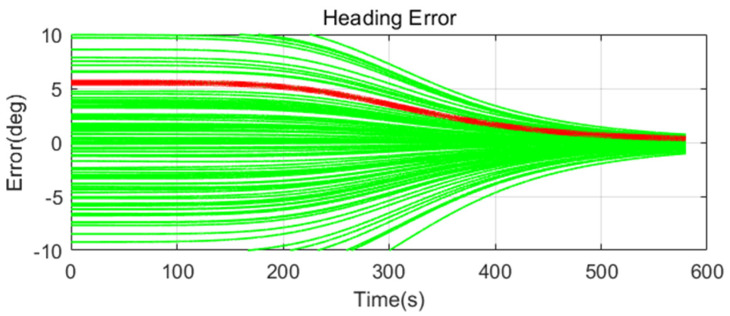
Heading error of alignment with CEKF (Case 1) in small size of movement.

**Figure 12:**
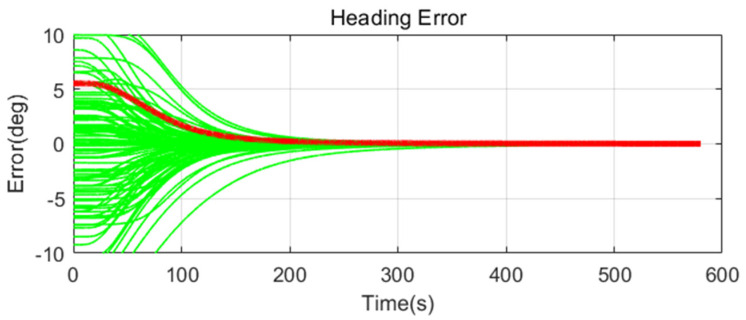
Heading error of alignment with Adaptive EKF (Case 2) in small size of movement.

**Figure 13:**
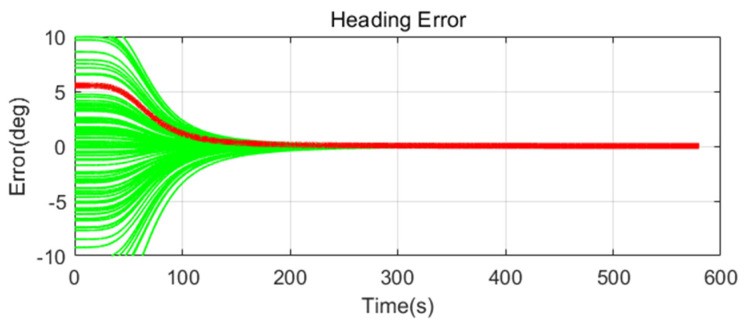
Heading error of alignment with learning-based EKF (Case 3) in small size of movement.

**Figure 14:**
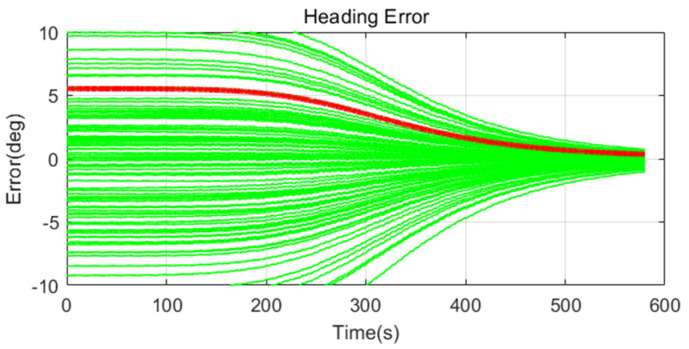
Heading error of alignment with CEKF (Case 1) in medium size of movement.

**Figure 15:**
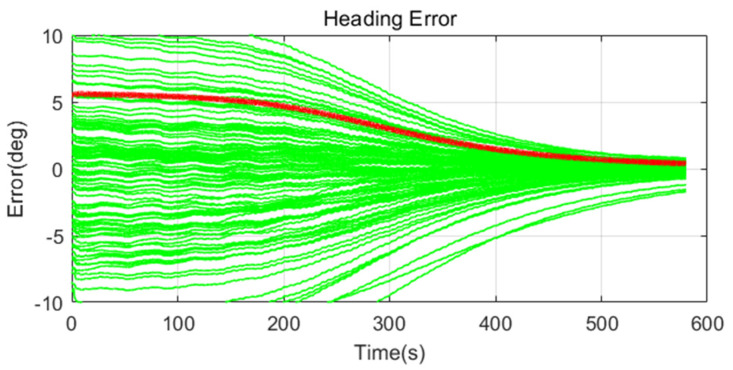
Heading error of alignment with Adaptive EKF (Case 2) in medium size of movement.

**Figure 16:**
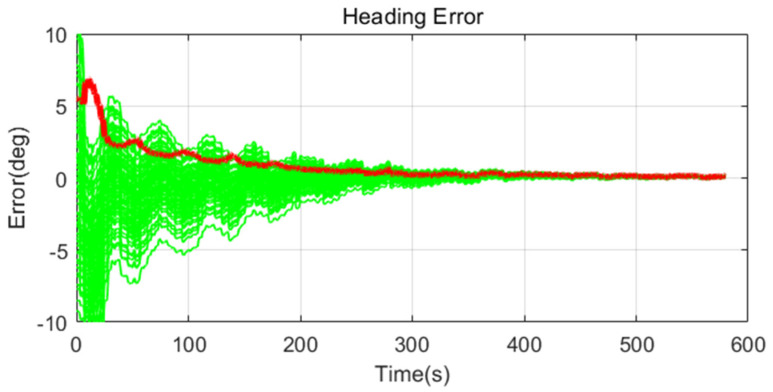
Heading error of alignment with learning-based EKF (Case 3) in medium size of movement.

**Figure 17:**
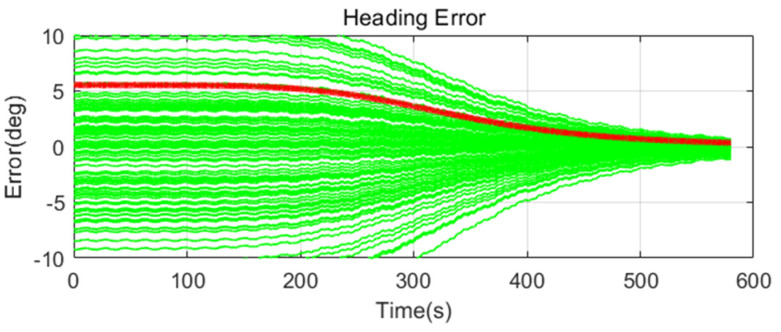
Heading error of alignment with CEKF (Case 1) in large size of movement.

**Figure 18:**
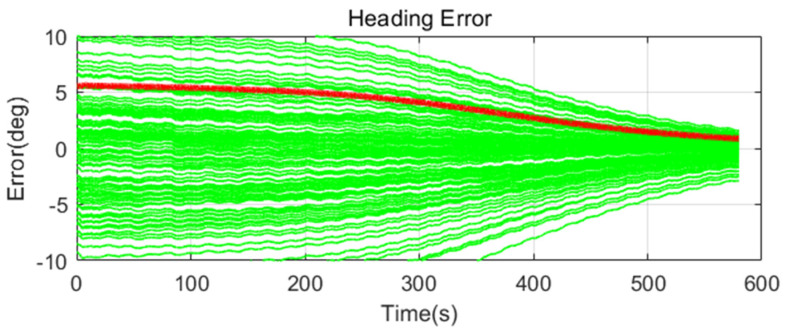
Heading error of alignment with Adaptive EKF (Case 2) in large size of movement.

**Figure 19:**
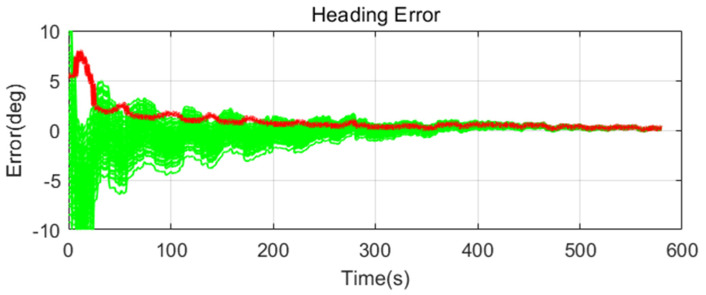
Heading error of alignment with learning-based EKF (Case 3) in large size of movement.

Simulation results are shown in Figure 11, Figure 12, Figure 13, Figure 14, Figure 15, Figure 16, Figure 17, Figure 18 and Figure 19 and Table 4, Table 5, Table 6, Table 7, Table 8 and Table 9. In the figures, the green lines show the results of 100 evolutions of Monte Carlo simulations with randomly set initial values. The red line is the RMS value of the result of 100 evolutions. Table 4, Table 6, and Table 8 show the RMS value of the error during the entire time for each case. Table 5, Table 7, and Table 9 show the RMS value of the last error for each case. In Table 4, Table 6, and Table 8, the times of convergence is defined as the time when the heading error (RMS) is within 20% of the initial heading error.

The figures show the heading angle error, which has a relatively large initial error and is the main goal of fine alignment. Moreover, the errors of the horizontal angle, such as roll and pitch, are summarized in the tables.

In Figure 11, Figure 12 and Figure 13 and Table 4 and Table 5, which are the cases where there is almost no disturbance, Case 1 (CEKF) converges very slowly. In contrast, Cases 2 (Adaptive EKF) and 3 (Learning-based EKF) converge quickly, and the last value shows a small error. This shows that the current mooring condition is almost static, thus the smaller is the measurement error covariance matrix, the better is the convergence and accuracy. In Cases 2 (Adaptive EKF) and 3 (Learning-based EKF), the measurement error covariance matrix value was adjusted to be small to adapt to the static environment, while Case 1 (CEKF) was maintained at a relatively large value in consideration of disturbance, thus reducing performance.

In Figure 14, Figure 15 and Figure 16 and Table 6 and Table 7, which are the cases where the size of the disturbance is medium, Cases 1 (CEKF) and 2 (Adaptive EKF) show that the alignment was performed despite the disturbance. However, in Case 1 (CEKF), it can be seen that the alignment was performed slowly by a relatively large measurement error covariance matrix designed for stability against the disturbance. In Case 2 (Adaptive EKF), the measurement error covariance matrix value was adaptively increased for the disturbance, but this matrix value is not considered to be optimized. In Case 3 (Learning-based EKF), since the optimization was performed to reduce the error by learning, the alignment was performed quickly and accurately despite the disturbance.

In Figure 17, Figure 18 and Figure 19 and Table 8 and Table 9, which are the cases where the size of the disturbance is relatively large, the overall result is similar to that of medium size of the movement, except that the performance is slightly worse as the size of the disturbance increase. In Case 2 (Adaptive EKF), the measurement error covariance matrix value was adaptively adjusted according to the size of the disturbance, but the performance was worse than in Case 1 due to the unoptimized value.

## 7. Conclusions

In this paper, we propose adaptive EKF and learning-based EKF to perform alignment using a Kalman filter in the mooring environment. Since the size of the wave in the mooring environment continuously changes with time and position, the conventional alignment method using a fixed measurement error covariance matrix has limitations. Therefore, as a method of adaptively adjusting the measurement error covariance matrix according to the disturbance, an alignment using an innovation-based adaptive EKF and a CNN-based learning method was applied.

The Monte Carlo simulation was performed by changing the initial errors and sensor errors and dividing the mooring condition into three types: small, medium, and large size of waves. 

As a result, in Case 1 (CEKF), the alignment was performed without the filter diverging despite the existence of disturbance. However, in all mooring conditions, alignment was performed very slowly regardless of the disturbance. In Case 2 (Adaptive EKF), a convergence of filter was fast and accurate when there was little disturbance, and it showed adaptive results that alignment was performed even when there was a disturbance. However, alignment was performed relatively slowly depending on the size of the disturbance because optimization was not applied. In Case 3 (Learning-based EKF), when there was little disturbance, alignment was high-speed and accurate, as if the alignment was performed in a static condition. Moreover, even if a disturbance occurs, it shows the best result regardless of the size of disturbance.

In the case of CNN-based alignment, it is necessary to learn various data in changing mooring conditions, but it enables fast and accurate alignment when performing alignment in a mooring. Therefore, it can be usefully applied to systems requiring fast and accurate alignment according to missions, such as surface vessel, ship, and submarine.

## Figures and Tables

**Table 1:** Convolution layer parameters.

Parameter	Value
$L_{out}$: length of output sequence	200
$L_{in}$: length of input sequence	200
P: length of padding	4
D: length of dilation	2
K: length of kernel (filter)	5
S: length of stride	1

**Table 2:** Learning parameters.

Sub Parameter	Value
Maximum epoch	200
Input dimension	6
Output dimension	3
Initial bias of CNN layer	$U\left(-\sqrt{\frac{1}{5C_{in}}},\sqrt{\frac{1}{5C_{in}}},\right)$
Initial weights of CNN layer	$U\left(-\sqrt{\frac{1}{5C_{in}}},\sqrt{\frac{1}{5C_{in}}},\right)$
Initial bias of fully connected layer	$U\left(-\sqrt{\frac{1}{5F_{in}}},\sqrt{\frac{1}{5F_{in}}},\right)$
Initial weights of fully connected layer	$U\left(-\sqrt{\frac{1}{5F_{in}}},\sqrt{\frac{1}{5F_{in}}},\right)$
Learning rate	0.00001
Gradient decay factor	0.000001
Optimizer	Adam
Drop ratio of learning rate	0.9

$C_{in}$, size of input channel; $F_{in}$, size of each input sample; *U*, uniform distribution.

**Table 3:** Simulation conditions.

	Sub Parameter	Value
**Sensor Error**	Gyro Bias	0.001°/hr (1σ, x/y/z axis)
	Gyro ARW	0.0005${}^{\circ}/\sqrt{\mathrm{hr}}$ (1σ, x/y/z axis)
	Accelerometer Bias	30 ug (1σ, x/y/z axis)
	Accelerometer Noise	20 ug (1σ, x/y/z axis)
**Initial** **Error**	Position	10 m (1σ, latitude/longitude axis)
	Velocity	0.1 m/s (1σ, north/east axis)
	Attitude (After Coarse Alignment)	Roll/Pitch 0.1° (1σ), Heading 5° (1σ)
**Wave Condition**	Attitude Amplitude	$A_{\phi }$ = 6°, $A_{\theta }$ = 8°, $A_{\psi }$ = 6°
	Attitude duration	$T_{\phi }$ = 7 s, $T_{\theta }$ = 5 s, $T_{\psi }$ = 6 s
	Velocity Amplitude	$A_{Pbx}$ = 0.3 m, $A_{Pby}$ = 1.0 m, $A_{Pbz}$ = 1.2 m
	Velocity duration	$T_{Pbx}$ = 7 s, $T_{Pby}$ = 6 s, $T_{Pbz}$ = 8 s
**Mooring Condition**	Small size of movement	10% of $\left[A_{\phi },A_{\theta },A_{Pbx},A_{Pby},A_{Pbz}\right]$
	Medium size of movement	50% of $\left[A_{\phi },A_{\theta },A_{Pbx},A_{Pby},A_{Pbz}\right]$
	large size of movement	100% of $\left[A_{\phi },A_{\theta },A_{Pbx},A_{Pby},A_{Pbz}\right]$
**Case**	Case 1	CEKF with fixed R (R = [0.1 m/s 0.1 m/s 0.1 m/s]^2^)
	Case 2	Adaptive EKF
	Case 3	Learning-based EKF
**Simulation Time**		600 s

**Table 4:** Attitude RMS error of alignment in small size of movement (100 evolutions).

Case	Roll (°)	Pitch (°)	Heading (°)	Convergence Times (s)
**CEKF**	0.01914	0.02767	3.88458	443.5
**Adaptive EKF**	0.00773	0.00698	1.81203	119.5
**Learning-based EKF**	0.00524	0.00517	1.78866	101.6

**Table 5:** Last attitude error of alignment in small size of movement (100 evolutions).

Case	Roll (°)	Pitch (°)	Heading (°)
**CEKF**	0.00235	0.00619	0.34707
**Adaptive EKF**	0.00159	0.00162	0.01579
**Learning-based EKF**	0.00159	0.00163	0.01252

**Table 6:** Attitude RMS error of alignment in medium size of movement (100 evolutions).

Case	Roll (°)	Pitch (°)	Heading (°)	Convergence Times (s)
**CEKF**	0.03147	0.03316	3.89140	444.3
**Adaptive EKF**	0.02995	0.03286	3.66340	435.6
**Learning-based EKF**	0.03479	0.04540	1.48509	146.7

**Table 7:** Last attitude error of alignment in medium size of movement (100 evolutions).

Case	Roll (°)	Pitch (°)	Heading (°)
**CEKF**	0.00220	0.02694	0.34814
**Adaptive EKF**	0.00197	0.02722	0.39209
**Learning-based EKF**	0.00186	0.02362	0.10507

**Table 8:** Attitude RMS error of alignment in large size of movement (100 evolutions).

Case	Roll (°)	Pitch (°)	Heading (°)	Convergence Times (s)
**CEKF**	0.09804	0.07607	3.91701	447.6
**Adaptive EKF**	0.09842	0.07782	4.06675	545.3
**Learning-based EKF**	0.09978	0.08042	1.54649	112.1

**Table 9:** Last attitude error of alignment in large size of movement (100 evolutions).

Case	Roll (°)	Pitch (°)	Heading (°)
**CEKF**	0.00879	0.10111	0.36676
**Adaptive EKF**	0.01031	0.10460	0.87981
**Learning-based EKF**	0.00963	0.09532	0.16823

## Appendix A

The system matrix for the Kalman filter is as follows:

$$F_{k}=\left[\begin{array}{cccc} F_{11} & F_{12} & F_{13} & 0_{3\times 3}\\ F_{21} & F_{22} & 0_{3\times 3} & F_{24}\\ 0_{3\times 3} & 0_{3\times 3} & 0_{3\times 3} & 0_{3\times 3}\\ 0_{3\times 3} & 0_{3\times 3} & 0_{3\times 3} & 0_{3\times 3} \end{array}\right]\;$$

$$F_{11}=\left[\begin{array}{ccc} \frac{v_{D}}{R_{m}+h} & 2\rho_{D}+2\Omega_{D} & -\rho_{E}\\ -2\Omega_{D}-\rho_{D} & \frac{v_{N}\tan L+v_{D}}{R_{t}+h} & 2\Omega_{N}+\rho_{N}\\ 2\rho_{E} & -2\Omega_{N}-2\rho_{N} & 0 \end{array}\right]$$

$$F_{12}=\left[\begin{array}{ccc} 0 & -f_{D} & f_{E}\\ f_{D} & 0 & -f_{N}\\ -f_{E} & f_{N} & 0 \end{array}\right]$$

$$F_{13}=C_{b}^{n}$$

$$F_{21}=\left[\begin{array}{ccc} 0 & \frac{1}{R_{t}+h} & 0\\ -\frac{1}{R_{m}+h} & 0 & 0\\ 0 & -\frac{\tan L}{R_{t}+h} & 0 \end{array}\right]$$

$$F_{22}=\left[\begin{array}{ccc} 0 & \Omega_{D}+\rho_{D} & -\rho_{E}\\ -\Omega_{D}-\rho_{D} & 0 & \Omega_{N}+\rho_{N}\\ \rho_{E} & -\Omega_{N}-\rho_{N} & 0 \end{array}\right]$$

$$F_{24}=-C_{b}^{n}$$

$$0_{3\times 3}=\left[\begin{array}{ccc} 0 & 0 & 0\\ 0 & 0 & 0\\ 0 & 0 & 0 \end{array}\right]$$

$$H=\left[\begin{array}{cccccccccccc} 1 & 0 & 0 & & & & & & & & & \\ 0 & 1 & 0 & & 0_{3\times 3} & & & 0_{3\times 3} & & & 0_{3\times 3} & \\ 0 & 0 & 1 & & & & & & & & & \end{array}\right]$$

where $R_{m}$ is the meridian radius of the curvature, $R_{t}$ is the transverse radius of the curvature in prime vertical, L is the latitude, h is the height, $C_{b}^{n}$ is the direction cosine matrix that converts from the body frame to the navigation frame, ${\left[\begin{array}{ccc} f_{N} & f_{E} & f_{D} \end{array}\right]}^{T}$ is the specific force in the NED navigation frame, ${\left[\begin{array}{ccc} \Omega_{N} & \Omega_{E} & \Omega_{D} \end{array}\right]}^{T}$ is the Earth’s rotation rate in the NED navigation frame, ${\left[\begin{array}{ccc} \rho_{N} & \rho_{E} & \rho_{D} \end{array}\right]}^{T}$ is the transport rate in the NED navigation frame [1], and H is a measurement matrix.
